# Genetics Evaluation Outcomes From an Academic Multidisciplinary Atypical Diabetes Program

**DOI:** 10.1210/jendso/bvaf091

**Published:** 2025-06-05

**Authors:** Colby L Chase, Marie-Louise Accardo, Veronica Greve, Elizabeth G Ames, Shane C Quinonez, Anthony Scott, Lauren Hipp, Jenna Damon, Wendy R Uhlmann, David T Broome, Maria C Foss-Freitas, Baris Akinci, Adam H Neidert, Goutham Narla, Elif A Oral, Kristen N Lee

**Affiliations:** Department of Internal Medicine, University of Michigan, Ann Arbor, MI 48109, USA; Department of Internal Medicine, University of Michigan, Ann Arbor, MI 48109, USA; Department of Internal Medicine, University of Michigan, Ann Arbor, MI 48109, USA; Department of Internal Medicine, University of Michigan, Ann Arbor, MI 48109, USA; Department of Internal Medicine, University of Michigan, Ann Arbor, MI 48109, USA; Department of Internal Medicine, University of Michigan, Ann Arbor, MI 48109, USA; Department of Internal Medicine, University of Michigan, Ann Arbor, MI 48109, USA; Department of Internal Medicine, University of Michigan, Ann Arbor, MI 48109, USA; Department of Internal Medicine, University of Michigan, Ann Arbor, MI 48109, USA; Department of Internal Medicine, University of Michigan, Ann Arbor, MI 48109, USA; Department of Internal Medicine, University of Michigan, Ann Arbor, MI 48109, USA; Department of Technological Research, Izmir Biomedicine and Genome Center & DEPARK, Dokuz Eylul University Health Campus, Izmir 35330, Turkey; Department of Internal Medicine, University of Michigan, Ann Arbor, MI 48109, USA; Department of Internal Medicine, University of Michigan, Ann Arbor, MI 48109, USA; Department of Internal Medicine, University of Michigan, Ann Arbor, MI 48109, USA; Department of Internal Medicine, University of Michigan, Ann Arbor, MI 48109, USA

**Keywords:** familial partial lipodystrophy, maturity onset diabetes of the young, monogenic obesity, genetic testing, genetic counseling

## Abstract

**Context:**

Rare monogenic conditions that predispose to diabetes can be misdiagnosed due to phenotypic overlap with more common conditions. Misdiagnosis can lead to ineffective, over-, or under-treatment. Specific genetic mechanisms can direct more precise treatment and facilitate clinical trial options. Recognition of characteristics of these conditions is necessary to facilitate high-yield referrals to genetics providers in order to improve diagnosis and treatment.

**Objective:**

Highlight clinical characteristics and diagnostic outcomes of patients undergoing genetics evaluation through a multidisciplinary Atypical Diabetes Program.

**Design, Setting, and Patients:**

Retrospective cohort review was completed for 87 patients referred to genetics from endocrinologists associated with the multidisciplinary Atypical Diabetes Program at a tertiary academic medical center between September 2019 and October 2022.

**Main Outcome Measure:**

Description of clinical characteristics of patients with a diagnostic or uncertain clinical genetic test result, as well as proportion of patients with these results.

**Results:**

Six patients (8.8%) had a pathogenic variant confirming diagnosis of lipodystrophy (4), monogenic diabetes (1), or monogenic obesity (1). Fifteen (22.0%) had a variant of uncertain significance, 5 of which correlated with their clinical features. As a result of genetics evaluation, all with a confirmed diagnosis had more precise treatment implemented and/or the opportunity to enroll in a clinical trial.

**Conclusion:**

Identification of rare genetic conditions predisposing to diabetes, enabled here through multidisciplinary genetics and endocrinology collaboration as part of the Atypical Diabetes Program, ultimately improves patient care. Endocrinologist attention to clinical features of these conditions is key to inform referral for genetics evaluation and testing.

Diabetes mellitus is a group of metabolic disorders characterized by hyperglycemia, which is due to defects in insulin secretion, insulin action, or both [1]. The most common forms of diabetes are type 1 and type 2. Atypical diabetes is a rare, often heritable group of conditions in which diabetes characteristics may differ from those with type 1 or type 2, additional health concerns are common, and different molecular etiologies exist. Most known atypical forms of diabetes are due to monogenic disorders, though polygenic or oligogenic contributions exist.

Monogenic diabetes was originally distinguished from a polygenic disorder in 1974 given identification of families with diabetes with a dominant mode of inheritance, and was subsequently studied in those who met the working criteria for maturity-onset type diabetes of the young (MODY) [2, 3]. The International Diabetes Federation Atlas reports that 10.5% of the adult population (20-79 years of age) has diabetes, with almost half unaware they are living with the condition. The International Diabetes Federation projection shows that, by 2045, 1 in 8 adults (approximately 783 million) will be living with diabetes, an increase of 46% [4]. Atypical forms of diabetes are estimated to affect greater than 1 in 1000 individuals in the general population, with the most common and underdiagnosed being lipodystrophy syndromes (familial partial lipodystrophy is the most underdiagnosed form), MODY, and nonsyndromic obesity, each with important implications for correct genetic diagnosis (Table 1). Given that diabetes is becoming increasingly more common, and with recent advances in genetic testing and precision medicine, it is likely that atypical forms of diabetes will be increasingly detected and novel forms described.

**Table 1.** Features, prevalence, and treatment for heritable conditions predisposing to diabetes

**FPLD**	
Clinical features	Associated with characteristic distribution of adipose tissue with loss of subcutaneous fat from extremities (predominantly lower limbs) along with accumulation in the face/trunk and sometimes dorsal region is seen. This is typically pubertal onset and more pronounced in individuals assigned female at birth [5]. Triad of insulin resistance with acanthosis nigricans, dyslipidemia (ie, hypertriglyceridemia and low high-density lipoprotein cholesterol), and nonalcoholic fatty liver disease ranging from simple steatosis to cirrhosis is common [5, 6]. Individuals may also present with cardiac disease (eg, hypertension, coronary artery disease, cardiomyopathy, arrhythmias), renal dysfunction (eg, proteinuria, membranoproliferative glomerulonephritis, focal segmental glomerulosclerosis), and reproductive dysfunction (eg, polycystic ovary syndrome, oligomenorrhea, hirsutism, preeclampsia). Subtypes are distinguished by genetic etiology [5]. *LMNA*-related FPLD (type 2) is the most well-phenotyped. *PPARG*-related FPLD (type 3) may be the most underdiagnosed given more subtle changes in adipose tissue but with metabolic comorbidities that are as severe or more severe than those of type 2 [7, 8]. Additional genes have been rarely identified in families (<5) with FPLD [5].
Prevalence	Genetic lipodystrophy has been reported in about 1000 individuals in the literature [9]. Molecular prevalence estimates have differed across studies, ranging from 1 in 7000 [10] to 12.4 per million [11].
Treatment	Focus on preventing and treating comorbidities with conventional therapies (metformin, insulin for diabetes mellitus, lipid-lowering drugs for dyslipidemia) [6]. Close cardiac monitoring given the high incidence of cardiac manifestations [12]. Experimental use of metreleptin considered for hypoleptinemic patients with severe metabolic derangements in the United States, with clinical trials available [5]. In Europe and Japan patients, those not responsive to conventional treatment can be prescribed metreleptin [5].
**MODY**	
Clinical features	Nonautoimmune, autosomal dominant diabetes mellitus onsets usually before age 35. Subtypes are genetically heterogeneous, with more than a dozen genes implicated [13]. Characteristics are atypical for type 1 diabetes mellitus, including absence of pancreatic islet antibodies, detectable C-peptide in the setting of hyperglycemia, endogenous insulin production beyond 3 to 5 years after onset, lack of ketoacidosis when insulin is omitted from treatment/low insulin requirement for effective treatment [13]. Often, but not always, individuals do not have risk factors for type 2 diabetes mellitus (significant obesity, acanthosis nigricans, hypertriglyceridemia) [13].
Prevalence	Greater than 1 in 1000 individuals in the general population and between 1% and 3% of those with diabetes are estimated to have MODY [14-16]. Studies to date mostly involve populations of European ancestry. In 1 study, around 4.5% of overweight/ obese adolescents diagnosed with type 2 diabetes mellitus were found to have MODY [17]. It has been reported to co-occur with diabetes mellitus type 1 or 2 [18].
Treatment	Treatment is highly dependent on genetic subtype: *HNF1A*-related MODY shows extreme sensitivity to sulfonylureas, while *GCK*-related MODY is not altered by medication and diabetes-related complications are rare [13]. Other genes account for 10% or fewer of MODY cases and may present with additional extra-pancreatic manifestations [13, 19, 20].
**Monogenic obesity**	
Clinical features	Severe obesity has a very early onset (≤ age 5), with or without syndromic features [21, 22]. Nonsyndromic forms may be accompanied by additional endocrine/metabolic complications but without other multisystem features [21]. Syndromic forms may be accompanied by additional endocrine/metabolic complications in addition to hypotonia, ophthalmic issues including retinal cone-rod dystrophy, cognitive impairment, developmental delay, sleep disruption, kidney disease, neurologic abnormalities including behavior/psychiatric, cardiovascular malformations, and other birth defects such as polydactyly [22].
Prevalence	Monogenic forms of obesity are estimated to account for 4% to 6% of severe obesity before age 6, with *LEPR* and *MC4R* genes being the most common etiologies [21, 23]. An estimated 5% to 6% of children and adults with obesity have a *MC4R* germline pathogenic [23, 24].
Treatment	Treatment implications vary by genetic subtype; research in this area is advancing rapidly [25-27].

Clinical features, prevalence, and treatment implications for major monogenic conditions predisposing to diabetes including FPLD, MODY, and monogenic obesity.

Abbreviations: FPLD, familial partial lipodystrophy; MODY, maturity-onset diabetes of the young.

As collaborations and genetic breakthroughs progressed, the ability to personalize treatment as well as identify at-risk relatives via cascade screening has increased. However, despite patient and provider interest, there are challenges to integrating genetics care into endocrinology via a multidisciplinary atypical diabetes program [28-31]. There is currently a paucity of genetic providers in a time of an increasing demand for genetic testing, rapidly advancing genetic testing technologies, and test results that require interpretation by both geneticists and endocrinologists teams for results to ultimately guide treatment [32, 33]. Various frameworks and practice resources to guide diabetes population screening to assess monogenic risk have been published [30, 32, 34]. Few centers have demonstrated successfully incorporating genetic diagnosis into routine diabetes care with posttest genetic counseling [35]. Therefore, endocrinologists’ attention to key clinical features suggestive of a genetic disorder is crucial to inform appropriate, high-yield referrals and assist in the diagnostic process.

The Atypical Diabetes Program at our institution leans on the historical strengths of both our endocrinology and genetics programs and research defining familial types of diabetes and lipodystrophy syndromes. In this retrospective cohort review, we describe diagnostic outcomes from this program with a focus on clinical characteristics of patients with a diagnostic or uncertain genetic test result, with the shared goal of facilitating more precise treatment for individuals with monogenic forms of diabetes.

## Materials and Methods

**Figure 1.**
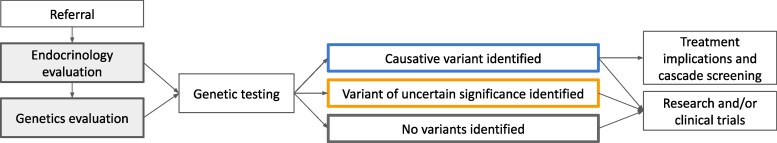
Flowchart depicting clinical care progression through the Atypical Diabetes Program, including endocrinology and genetics evaluations with the potential for more precise treatment and/or research trial eligibility.

Clinical evaluations were facilitated through the Atypical Diabetes Program. This program was established as a partnership between endocrinology and the medical genetics clinic, in which the Atypical Diabetes Genetics (ADG) clinic was formed to streamline genetics evaluation of patients referred by a treating endocrinologist with an interest in atypical forms of diabetes (Fig. 1). Patients were referred by endocrinology providers to the ADG clinic if they had clinical features strongly suggestive of an established monogenic condition (eg, clinical lipodystrophy, early-onset β-cell failure without autoimmunity, early-onset obesity and diabetes together), especially if there was a family history of similar features consistent with an autosomal dominant inheritance pattern, multisystem features (another system impacted in addition to glucose abnormalities), or research on genetic testing requiring clinical confirmation. Patients referred to the ADG clinic were eligible for expedited clinic appointments with the option to coordinate a visit with their endocrinology provider, with the aim of increasing ease and uptake of scheduling the evaluation.

**Figure 2.**
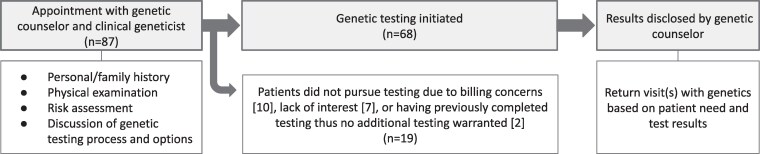
Flowchart depicting clinical evaluations through the Atypical Diabetes Genetics clinic, including aspects of genetics evaluation and number of patients pursuing or declining genetic testing.

Clinical evaluations in the ADG clinic occurred with a genetic counselor and a clinical geneticist (Fig. 2). An initial evaluation in the ADG clinic included gathering personal and family medical history as well as a physical examination focused on features of syndromic conditions. Clinical evaluations, and genetic testing if pursued, were billed through patient insurance. There were no specific criteria in place for offering genetic testing; all patients were given the option to pursue testing relevant to their clinical history and features based on their endocrine or other diagnoses. Genetic testing was initiated through clinical genetic testing laboratories, with variant interpretation performed by the testing laboratory; tests ordered included a monogenic diabetes gene panel, a monogenic obesity gene panel, a lipodystrophy gene panel, whole-exome sequencing, or other phenotype-driven testing. Custom panels curated by the ADG providers were developed through a review of monogenic diabetes, monogenic obesity, and lipodystrophy genes; a review of commercial laboratory offerings; and a literature search of candidate genes. Additional genetic testing beyond the customized/curated atypical diabetes panels was sometimes conducted based on clinical presentation, particularly when incidental findings during the genetic evaluation suggested the presence of another genetic condition. Whole-exome sequencing was ordered when both atypical diabetes and nonendocrinology genetic conditions (without availability of a targeted gene panel) were being evaluated or when targeted atypical diabetes gene panels were nondiagnostic with high clinical suspicion remaining. Results of clinical genetic testing were disclosed by a genetic counselor with additional genetics appointments coordinated based on patient need and genetic testing results.

We performed a retrospective cohort review of patients referred to the ADG clinic as part of the Atypical Diabetes Program over a 3-year period (August 2019-October 2022 after institutional review board review; HUM00181262). Data relating to demographics (age, sex designated at birth), referral indication, previous research-based genetic testing results, differential diagnosis following genetics evaluation, phenotype information [body mass index (BMI), metabolic abnormalities, age of onset], and genetic testing information (indication, test, outcome) was collected and managed using REDCap electronic data capture tools hosted at the University of Michigan [36, 37]. This manuscript focuses on the outcomes of genetic investigations pursued through clinical care and not research endeavors that followed the clinical evaluations. Clinical evaluation outcomes are presented here as descriptive statistics and case vignettes, which provide key clinical examples of patient results and impact on care.

## Results

### Demographics and Genetics Evaluation Outcomes

Eighty-seven patients were evaluated, and 68 (78.2%) patients pursued genetic testing. Of the 68 patients who had genetic testing, 49 (72.1%) patients identified as female, and the mean patient age at evaluation was 41.2 years (range 12-78). Twenty-nine patients (42.6%) were tested for monogenic obesity (syndromic and/or nonsyndromic); 29 for lipodystrophy (42.6%); 16 for monogenic diabetes (23.5%); 7 for familial dyslipidemia (10.3%); and 18 for other diagnoses including but not limited to hereditary cancer predisposition, multisystem health issues, and/or congenital anomalies, hypogonadotropic hypogonadism, hearing loss, immune dysregulation, or developmental delay (26.5%) (Table 2). Twenty-one patients (30.9%) were tested for more than 1 indication (endocrinologic or nonendocrinologic) listed previously given clinical features raising suspicion for more than 1 condition. Nearly 80% of patients had multigene panel testing related to the suspected indication while others had exome, chromosomal microarray, or multiple tests. Reasons for not testing included lack of insurance coverage or high out-of-pocket cost (10), lack of interest in testing (7), or previously completed testing, thus no additional testing was warranted (2).

**Table 2.** Genetic testing

**Genetic testing indication** *^a^*	**Number of patients (%)**
Monogenic obesity	29 (42.6)
Lipodystrophy	29 (42.6)
Monogenic diabetes	16 (23.5)
Familial dyslipidemia	7 (10.3)
Other diagnoses	18 (26.5)
Hereditary cancer predisposition	5 (7.4)
Multisystem health issues/congenital anomalies	3 (4.4)
Hypogonadotropic hypogonadism	2 (2.9)
Hearing loss	2 (2.9)
Immune dysregulation	2 (2.9)
Developmental delay	2 (2.9)
Adrenomyeloneuropathy	1 (1.5)
Overgrowth syndromes	1 (1.5)
Alport syndrome	1 (1.5)
Muscular dystrophy	1 (1.5)
Multiple listed indications	21 (30.9)
**Testing strategy** *^a^*	
Condition-specific test or multigene panel only	54 (79.4)
Whole-exome sequencing (first-line)	10 (14.7)
With mitochondrial genome analysis	7 (10.3)
Multigene panel (first-line) followed by whole-exome sequencing with mitochondrial analysis	2 (2.9)
Chromosomal microarray (first-line) followed by whole-exome sequencing	1 (1.5)
Concurrent chromosomal microarray and multigene panel	1 (1.5)
**Genetic testing yield for select indications**	
Monogenic obesity	1/29 (3.4)
Lipodystrophy	4/29 (13.8)
Monogenic diabetes	1/16 (6.3)

Genetic testing indication, strategy, and yield for select phenotypic presentations evaluated.

^a^n = 68.

Results presented here focus on the results of testing for monogenic obesity, lipodystrophy, monogenic diabetes, and familial dyslipidemia. Six (8.8%) patients had a diagnostic result for lipodystrophy (4), monogenic diabetes (1), or monogenic obesity (1). Fifteen (22.0%) patients had variants of uncertain significance identified on testing for these conditions, and 4 (5.9%) had results showing incidental carrier status that do not provide a diagnosis (Table 3). The following case vignettes highlight cases with diagnostic results (pathogenic or likely pathogenic variants) or potentially diagnostic results (variants of uncertain significance in genes where the patient presentation is highly consistent with the disorder associated with that gene).

**Table 3.** Variants of uncertain significance, or pathogenic variants related to carrier status/nonendocrine symptoms

Patient (age, sex assigned at birth)	Brief description of symptoms	VUS Heterozygous, unless specified	Pathogenic or likely pathogenic variant(s) related to carrier status *Heterozygous, unless specified*	Pathogenic variant(s) related to non-endocrine symptoms *Heterozygous, unless specified*
33y, male	Reported muscle weakness and wasting/atrophy, dysphagia and hoarse speech, and tremor Intermittent lymphadenopathy, recurrent viral infections, sinusitis, bronchitis, intermittent low-grade fever, and recurrent rashes Presyncopal episodes, cardiac palpitations, and chronic fatigue; unintentional weight loss and mildly C5 and C1Q complement deficiency	*MFN2* gene (c.1100A > G, p.Gln367Arg)	NA	NA
42y, female	Accumulation of adipose tissue in trunk and neck noted after pregnancies, decrease of adipose tissue in arms and legs Reports that this has always been her body type with weight loss difficult to achieve despite being fairly active Diagnosed with diabetes mellitus in early 40s and has diagnoses of nonalcoholic fatty liver disease, hypertension, PCOS, and hirsutism No similar family history	NA	*PSMB8* gene, (c.163C > T, p.Gln55*) Associated with AR Nakajo-Nishimura syndrome, characterized by infantile- or childhood-onset recurrent fevers, skin manifestations (nodular erythema, rash), joint contractures, and lipodystrophy especially in the upper body	NA
59y, female	Atypical distribution of adipose tissue with onset in her 20s, hypertriglyceridemia diagnosed in her early 40s Primary aldosteronism with hypokalemia, bilateral adrenal adenomas, hyperparathyroidism Muscle cramping, tightening, and exercise-induced muscle fatigue with onset in her 40s at the time of statin initiation Mild sensorineural hearing loss bilaterally, audiogram notable for “cookie bite” hearing loss	*WRN* gene (c.201A > G, p.E67E=) *APOB* gene (c.1075G > A, p.Ser359Gly)	NA	*GJB2* gene (c.23C > T, p.T8M) Associated with autosomal dominant and recessive forms of nonsyndromic hearing loss
27y, male	Grade III obesity with weight gain and food-seeking behaviors starting in childhood History of developmental delay and other childhood-onset concerns (abnormal rib cage, hernia, undescended testes, macrocephaly, severe constipation, strabismus/severe myopia) No similar family history of similar developmental delay or intellectual disability; history of grade III obesity in brother and mother	*RNF135* gene (c.176G > A, p.Cys59Tyr) *IFT172* gene (c.650G > A, p.Arg217Gln) *PCNT* gene *(*c.4675A > T, p.Met1559Leu)	*LEPR* gene (c.3G > A, p.Met1?) Associated with AR obesity and hypogonadotropic hypogonadism due to leptin receptor deficiency	*CDK13* gene (c.2142_2150del, p.T715_G717del) Associated with intellectual disability, developmental delay, feeding difficulties in infancy, structural cardiac defects, and seizures with other reported features including severe constipation, strabismus, and macrocephaly
21y, female	Changes in adipose tissue and other characteristics starting in childhood and becoming more noticeable by adolescence: loss in the face/cheeks, limited breast development, limited fat in abdomen, nose becoming larger and curving downward, lack of fat in fingertips, flat feet Dense deposit glomerulonephritis (autoimmune with low C3) diagnosed around age 13, with eventual long-term steroid treatment and subsequent dilated cardiomyopathy	*GPIHBP1* gene (c.475G > A, p.Gly159Ser)	*HADHA* gene (c.1528G > C, p.E510Q) Associated with AR long-chain 3-hydroxyacyl-CoA dehydrogenase deficiency, a fatty acid oxidation disorder	NA
47y, male	Diabetes diagnosed in his late 30s with positive GAD65 antibodies consistent with latent autoimmune diabetes in adults	*PAX4* gene (c.361C > T, p.Arg121Trp)	NA	NA
43y, male	Hypertriglyceridemia diagnosed around age 7 or 8, has been >6000 mg/dL Type 2 diabetes diagnosed at age 21; at age 34 found to have elevated GAD65 antibodies consistent with type 1 diabetes Had an episode of acute pancreatitis at age 35; also found to have steatohepatitis and borderline splenomegaly Diagnosed with chronic inflammatory demyelinating polyneuropathy at age 35 and Sjogren's syndrome at age 36 History of recurrent pericarditis Physical examination notable for “central obesity, tall stature, large trunk, thin relatively muscular limbs”	*APOB* gene (c.1166A > C, p.Gln389Pro)	NA	NA
30y, female	Type 2 diabetes diagnosed at age 22 Hypertriglyceridemia diagnosed at age 22 (highest reported 1500 mg/dL) Body habitus of truncal obesity with thin muscular extremities Significant family history of cancer (maternal and paternal), notably with report of relatives with Lynch syndrome Height 5′ 7″, family history of heart attack and valve disease requiring surgical correction, no known arterial aneurysm or lens dislocation	*FBN1* gene (c.250A > G, p.Ile84Val) *BRCA2* gene (c.8739C > G, p.Asp2913Glu)	NA	NA
41y, female	Type 1 diabetes since 14 months of age, continues to have severe insulin resistance with high insulin antibody testing Hyperlipidemia diagnosed in adolescence. Postpubertal obesity with some but not significant response to gastric sleeve surgery	NA	*LMF1* (c.733C > T, p.Gln245*) Associated with AR combined lipase deficiency, characterized by severe hypertriglyceridemia	NA
24y, male	Has always been in the upper limit for height and weight; increased weight gain around age 10 to 11 Bone abnormalities including reported “bowed legs” noted around age 13 to 14 as well as severe kyphoscoliosis and spinal stenosis diagnosed at age 18 Reported diagnosis of platelet dense granule disorder at age 5; hematology evaluation at age 22 with no concern for bleeding disorder Hypertension and type 2 diabetes diagnosed at age 13 or 14. Physical examination at age 24 consistent with morbid obesity (56.88 kg/m^2^) with large lipomatous areas and dorsal hump with kyphoscoliosis	*BLK* gene (c.76C > G, p.L26V) *PPPR25D* gene (c.479 G > A, p.R160H)	NA	NA
28y, male	Type 2 diabetes at age 25, with BMI of 21.02 kg/m^2^. NAFLD with mild fibrosis; reported previously elevated cholesterol and triglycerides treated with statin	*WFS1* gene (c.1744 G > A, p.V582M)	NA	NA
43y, male	Alport syndrome diagnosed via renal biopsy at age 9; manifestations include renal disease, sensorineural hearing loss, and corneal erosions Childhood-onset hyperlipidemia reported to have been inversely correlated with proteinuria levels Myocardial infarction at age 32 treated with coronary artery bypass Developed diabetes following renal transplant at age 36 History of autism spectrum disorder and 6 lifetime colonic adenomas	*MSH6* gene (c.2440A > G, p.K814Q) *MT-RNR1* gene (m.1042T > A [homoplasmic])	NA	*COL4A5* gene (c.3374-1G > A, splice site [hemizygous]) Associated with X-linked Alport syndrome characterized by early-onset renal failure, hearing loss, and eye abnormalities
51y, male	Type 1 diabetes at age 23 with high GAD65 antibodies with family history of type 1 diabetes in his daughter and nephew Gastrointestinal stromal tumor diagnosed at age 43 and high-grade dysplastic tubular adenoma diagnosed at age 49	*SMARCA4* gene (c.4864 + 5G > A, intronic)	NA	NA
49y, female	Suspected lipodystrophy with consistent body habitus, decreasing muscle and fat in extremities around age 30 with increasing stomach growth around age 35 Fatty liver disease diagnosed at age 14. Hyperlipidemia with significantly elevated triglycerides since age 23 (up to the 900s). Type 2 diabetes diagnosed around age 32 to 33 Neuropathy with recent spread from her right lower extremity to right upper extremity. Chronic right leg pain with recent spread to include left leg and right arm. Braces on her legs in childhood for an unknown reason. Stroke at age 47 and subsequent PFO/ASD closure History of frequent/recurrent nosebleeds in childhood, sometimes requiring nasal packing/cautery. Child with clinically diagnosed hereditary hemorrhagic telangiectasia	*APOB* gene (c.8550T > G, p.I2850M)	NA	NA

Genetic test results for 14 patients, in addition to those highlighted in case vignettes, with VUS or pathogenic variants related to carrier status (ie, identification of a single heterozygous pathogenic variant in a gene associated with an AR condition) or a nonendocrine condition. These are considered nondiagnostic results/not suspected to be related to the patient's clinical presentation based on current knowledge.

Abbreviations: AR, autosomal recessive; BMI, body mass index; NA, not applicable; NAFLD, nonalcoholic fatty liver disease; PCOS, polycystic ovary syndrome; PFO/ASD, patent foramen ovale/atrial septal defect; VUS, variants of uncertain significance.

### Case Vignettes: Diagnostic Results

#### Familial partial lipodystrophy type 2—*LMNA* gene (c.1445G > A, p.Arg482Gln)

**Figure 3.**
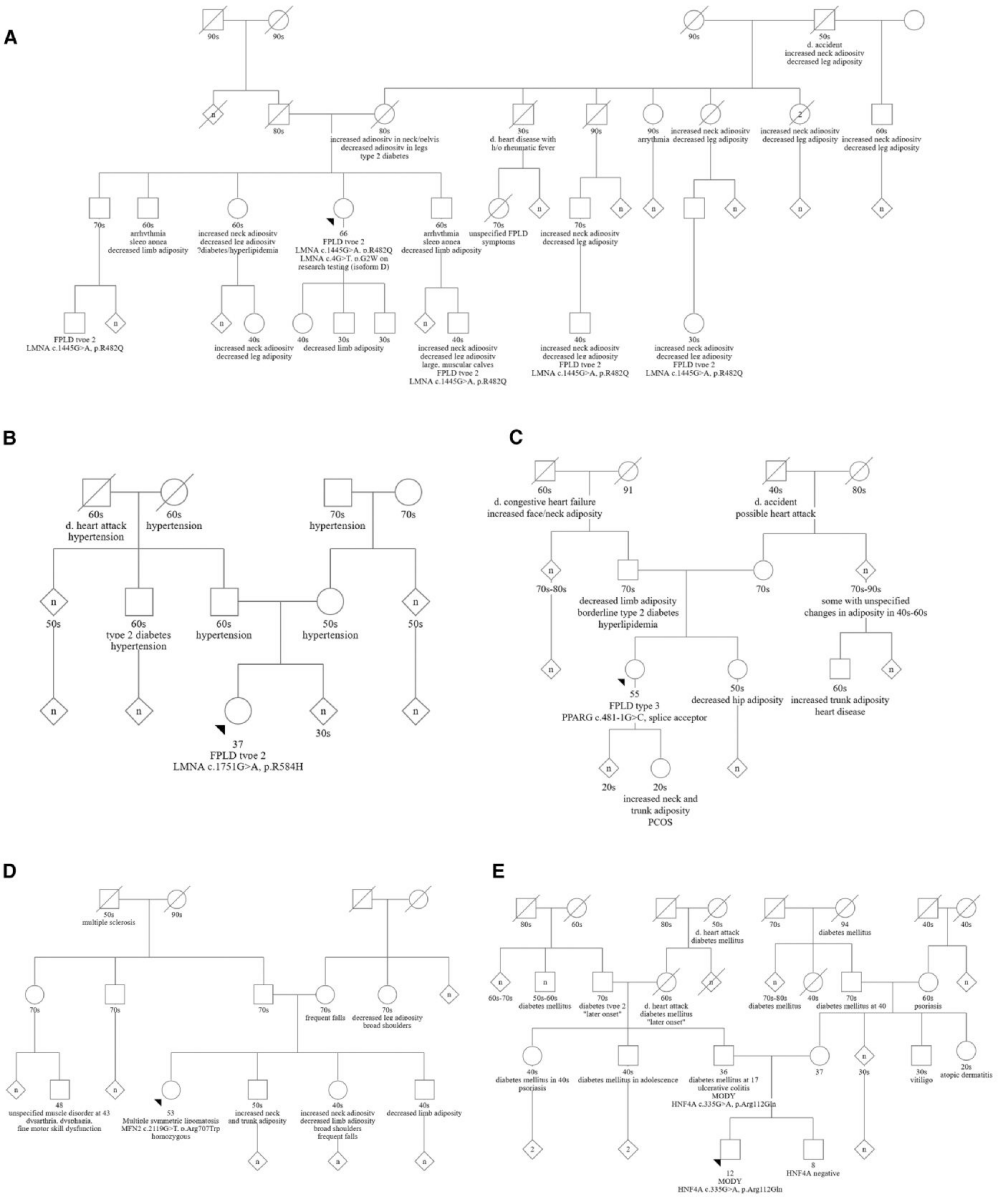
Pedigrees from 5 patients with confirmed genetic diagnoses following Atypical Diabetes Genetics evaluation. (A) Heterozygous pathogenic *LMNA* gene variant (c.1445G > A, p.R482Q) consistent with diagnosis of familial partial lipodystrophy type 2. (B) Heterozygous likely pathogenic *LMNA* gene variant (c.1753G > A, p.Arg584His) consistent with diagnosis of familial partial lipodystrophy type 2. (C) Heterozygous likely pathogenic *PPARG* gene variant (c.481-1G > C, splice site) consistent with diagnosis of familial partial lipodystrophy type 3. (D) Homozygous pathogenic *MFN2* gene variant (c.2119G > T, p.Arg707Trp) consistent with diagnosis of Charcot-Marie-Tooth type 2A disease and multiple symmetric lipomatosis (Madelung's disease). (E) Heterozygous pathogenic *HNF4A* gene variant (c.335G > A, p.Arg112Gln) consistent with diagnosis of maturity onset diabetes of the young type 1.

A 66-year-old female presented with atypical body fat distribution and metabolic symptoms. After puberty, she experienced loss of adipose tissue in her upper arms and calves, and in her 50s she experienced accumulation in her neck and pubic region. Her medical history was significant for controlled hypertension with onset in her 50s to 60s, left bundle branch block, intermittent elevated triglycerides, and hepatic steatosis, among other medical diagnoses. She reported a family history of multiple first-degree, second-degree, and further removed relatives with atypical distribution of adipose tissue, as well as individuals with cardiac disease, diabetes, and/or other health concerns (Fig. 3A).

Previous research-based genetic testing reported 2 *LMNA* gene variants: (c.1445G > A, p.R482Q; NM_170707.4) and (c.4G > T, p.G2W; NM_001257374, isoform D). p.G2W corresponds to a deep intronic region of the major *LMNA* gene transcripts encoding isoforms A and C and thus is outside of the reportable range for most clinical laboratories and has not been reported in the medical literature. The patient denied a history of contractures in early childhood or progressive muscle weakness/wasting, thus a diagnosis of Emery-Dreifuss muscular dystrophy (caused by biallelic *LMNA* pathogenic variants) was not suspected. Clinical analysis of the *LMNA* gene via next-generation sequencing confirmed the presence of the heterozygous pathogenic missense variant p.R482Q in the patient, supporting the diagnosis of familial partial lipodystrophy (FPLD) type 2.

Metreleptin or other leptin agonists were suggested in the context of a clinical trial, but she did not meet all trial inclusion criteria given her adequate metabolic control and is being followed clinically. She was already established with a cardiologist, and diagnostic results were relayed to her clinician team for more frequent follow-up. Previously undiagnosed relatives underwent cascade screening, enabling diagnosis in family members.

#### FPLD type 2—*LMNA* gene (c.1753G > A, p.Arg584His)

A 32-year-old female presented with body habitus suggestive of partial lipodystrophy with limb and pelvis lipoatrophy beginning in childhood. At puberty, she developed fat accumulation in the face, neck, perineal and intra-abdominal areas, and dorsocervical region, as well as calf muscularity. She had a BMI of 39.79 kg/m^2^, and she had difficulty maintaining weight loss. She was monitored for prediabetes and had a history of dyslipidemia. In addition, she had polycystic ovarian syndrome and hypertension diagnosed at 19 as well as hepatic steatosis. She reported a strong family history of hypertension (Fig. 3B).

A custom multigene panel including next-generation sequencing with copy number variation analysis of genes *AGPAT2, AKT2, BANF1, BSCL2, CAV1, CIDEC, FBN1, KCNJ6, LIPE, LMNA, MFN2, PCYT1A, PIK3R1, PLIN1, POLD1, PPARG, PSMA3, PSMB4, PSMB8, PSMB9, PTRF, SPRTN, TBC1D4, WRN,* and *ZMPSTE24* identified a likely pathogenic heterozygous missense variant in the *LMNA* gene (c.1753G > A, p.Arg584His), consistent with a diagnosis of FPLD type 2.

This patient was informed of a clinical trial for FPLD; however, she did not display sufficient metabolic abnormalities to qualify for this trial. Echocardiogram and cardiology follow-up were recommended given the reported association between *LMNA* gene pathogenic variants and cardiomyopathy, and she is now under the care of a local cardiologist (specific results not available). First-degree relatives were recommended to consider genetics evaluation.

#### FPLD type 3—*PPARG* gene (c.481-1G > C, splice site)

A 55-year-old female presented with atypical body fat distribution accompanied by endocrine comorbidities. Specifically, she reported having muscular calves and broad shoulders since childhood; increased fat deposition in her trunk, face, and neck in her mid-40s; and most recently a loss of fat in her fingers and toes. On physical examination, she also had loss of fat in the hips and buttocks. She was diagnosed with dyslipidemia at age 35, diabetes at age 45 (treated with insulin), and hepatic steatosis at age 55. She reported a family history of multiple first-, second-, and third-degree relatives with atypical distribution of adipose tissue, as well as individuals with cardiac disease, diabetes, and/or other health concerns (Fig. 3C).

A custom multigene panel including next-generation sequencing with copy number variation analysis of genes *AGPAT2, AKT2, BANF1, BSCL2, CAV1, CIDEC, FBN1, KCNJ6, LIPE, LMNA, MFN2, PCYT1A, PIK3R1, PLIN1, POLD1, PPARG, PSMB8, PTRF, SPRTN, TBC1D4, WRN,* and *ZMPSTE24* identified a likely pathogenic heterozygous splice acceptor variant in the *PPARG* gene (c.481-1G > C, splice site) consistent with a diagnosis of FPLD type 3.

Pioglitazone was started following her diagnosis with a reported slight improvement in her hemoglobin A1c (HbA1c). She also participated in a clinical trial testing the efficacy of a novel leptin receptor agonist in FPLD. First-degree relatives were recommended to consider genetics evaluation.

#### Multiple symmetric lipomatosis (Madelung's disease)—*MFN2* gene (c.2119G > T, p.Arg707Trp)

A 53-year-old female presented with atypical body fat distribution accompanied by endocrine comorbidities. Dating back to childhood, she reported increased facial adiposity, which increased in adolescence. At puberty, she also developed fat accumulation in the lower abdomen and underwent liposuction twice at ages 18 and 19, while her legs remained thin with defined-appearing muscles. In her early 20s, she continued to accumulate fat tissue in the face, upper back, and shoulders. She has a history of hypertriglyceridemia and elevated liver function tests. She has had lipomas in her shoulder region. Her reproductive history is notable for dysmenorrhea, which improved with the use of oral contraceptive pills. She denied a history of insulin resistance, diabetes, or acanthosis nigricans.

The patient additionally reported difficulty running and walking down steps beginning at age 49 to 50, as well as difficulty standing up from a seated position. She described a sensation of “stiffness” in her legs and has noted fasciculations in her legs, weakness in her feet, tripping at times, and dropping objects. A neurology evaluation was significant for EMG consistent with axonal neuropathy and clinical impression suggestive of inherited neuropathy given her family history, notable for frequent falls in her mother and sister (Fig. 3D).

A custom multigene panel including next-generation sequencing with copy number variation analysis of genes *AGPAT2, AKT2, BANF1, BSCL2, CAV1, CIDEC, FBN1, KCNJ6, LIPE, LMNA, MFN2, PCYT1A, PIK3R1, PLIN1, POLD1, PPARG, PSMA3, PSMB4, PSMB8, PSMB9, PTRF, SPRTN, TBC1D4, WRN,* and *ZMPSTE24* identified a germline homozygous *MFN2* pathogenic variant (c.2119G > T, p.Arg707Trp) consistent with a diagnosis of Charcot-Marie-Tooth type 2A. In addition, patients homozygous for this variant had been reported in the medical literature with a clinical presentation termed multiple symmetric lipomatosis (Madelung's disease), involving lipomatous masses and lipoatrophy [38]. She is participating in a clinical trial for this condition.

#### Maturity-onset diabetes of the young type 1—*HNF4A* gene (c.335G > A, p.Arg112Gln)

A 12-year-old male presented with a personal history of Crohn's disease diagnosed at age 10, chronic recurrent multifocal osteomyelitis diagnosed at age 12, and incidentally identified hyperglycemia diagnosed as diabetes type 1 despite normal IA-2 and glutamic acid decarboxylase antibodies. Pediatric immunohematology evaluation did not identify a specific underlying diagnosis; a history of recurrent infections was denied. His family history was remarkable for diabetes mellitus, notably young-onset disease in his father and paternal uncle, as well as paternal and maternal relatives with various autoimmune diseases (Fig. 3E).

Whole-exome sequencing with parental samples submitted for comparative purposes was pursued given the possibility of multiple genetic diagnoses. Results identified a pathogenic variant in the *HNF4A* gene (c.335G > A, p.Arg112Gln) in both the patient and his father supportive of a diagnosis of maturity-onset diabetes of the young (MODY) type 1.

Results have impacted medical care for the family; the adolescent's HbA1c will be monitored, and he will initiate sulfonylurea if this rises above 7%; his father sought to re-establish care with endocrinology for his diabetes management. The family was encouraged to share this information with paternal relatives, especially those with a known diagnosis of diabetes. While genome-wide association studies have identified common polymorphisms in the *HNF4A* gene associated with ulcerative colitis, insufficient evidence is available to determine whether rare deleterious variants in this gene increase risk for ulcerative colitis or other autoimmune disorders [39].

#### Prader-Willi syndrome—abnormal methylation of 15q11.2-15q13.1 genomic region

A 35-year-old female presented with a longstanding suspected diagnosis of Prader-Willi syndrome with a history of hypotonia in infancy with normal birth weight, persistent weight gain starting at 18 months of age, and hyperphagia with food-seeking behaviors. Other diagnoses included hypogonadism secondary to hypothalamic dysfunction, hypertension, diabetes, and acanthosis nigricans. Her BMI was 62.73 kg/m^2^ at the time of genetics evaluation. There was no reported family history of similar concerns. Genetic testing was requested to confirm the diagnosis given the recently recognized heterogeneity in patients with a clinical diagnosis of Prader-Willi syndrome and the need for molecular diagnosis for eligibility for certain clinical trials.

Methylation-specific multiplex ligation-dependent probe amplification for Angelman/Prader-Willi syndrome showed abnormal methylation of the 15q11.2-15q13.1 genomic region, consistent with maternal uniparental disomy or imprinting defect, and normal copy number analysis of this region, confirming the clinical diagnosis of Prader-Willi syndrome. She was offered clinical trial participation but declined due to current logistical considerations.

### Case Vignettes: Variants of Uncertain Significance Correlating to Phenotype

#### Nonsyndromic obesity—*SH2B1* gene (c.2101G > A, p.Val701Met)

A 34-year-old female presented with morbid obesity and a history of early-onset obesity with increased appetite and food-seeking behaviors reported starting around age 3 or 4; development was otherwise reported to be normal. Weight gain has been mostly refractory to lifestyle interventions, medication management, and gastric bypass, which resulted in weight loss but with a plateau above the target weight followed by a continued increase. Her highest weight was 560 lbs. Her history is also significant for hypertension and prediabetes with insulin resistance.

A custom multigene panel including next-generation sequencing with copy number variation analysis of genes *KSR2, LEP, LEPR, MC4R, NR0B2, NTRK2, PCSK1, POMC, SH2B1, SIM1,* and *UCP3* identified a variant of uncertain significance in the *SH2B1* gene (c.2101G > A, p.Val701Met). Pathogenic variants in this gene have been identified in individuals with obesity and hyperinsulinemia. One study also described variable neurodevelopmental concerns including speech and language delays or aggressive behavior [40]. The patient has been treated in a trial of the experimental drug setmelanotide, meeting eligibility criteria given in silico predictive characteristics of this variant as established by the manufacturer.

#### Nonsyndromic obesity—*MC4R* gene (c.235A > G; p.Met79Val)

A 20-year-old female presented with difficulty losing weight. She reported starting to gain excess weight at age 8 refractive to increased physical activity. Menarche occurred at age 12 to 13, and menstrual bleeding has been continuous. In adolescence, she was diagnosed with hypothyroidism and started on levothyroxine. She was later diagnosed with a platelet storage pool disorder. She was diagnosed with neurocardiogenic syncope by tilt test. She denied a history of developmental delay, failure to thrive, hypotonia, autism spectrum disorder, excessive dental cavities, insulin resistance, diabetes, acanthosis nigricans, polycystic ovarian syndrome, hirsutism, hearing or vision concerns, and kidney disease. At the time of the genetics evaluation, her height was 5′ 11.5″ and BMI was 33.20 kg/m^2^.

A custom multigene panel including next-generation sequencing with copy number variation analysis of genes *BDNF, CEP19, DYRK1B, GNAS, KSR2, LEP, LEPR, MC4R, MRAP2, NPY, NR0B2, NTRK2, PCSK1, POMC, SH2B1, SIM1,* and *UCP3* identified a germline heterozygous variant of uncertain significance in the *MC4R* gene (c.235A > G, p.Met79Val). Segregation analysis was pursued and the variant confirmed to be inherited from her mother, who was overweight. The *MC4R* gene has been associated with susceptibility to nonsyndromic obesity.

#### MODY type 2—*GCK* gene (c.679 + 3A > T, splice site)

A 35-year-old female presented with atypical diabetes. She reported excess weight gain beginning at age 8 despite being “very active” with HbA1c at this time reportedly 6.8%. Initial genetics evaluation was conducted at age 17, at which time she recalls analysis of the *HNF1A* gene was negative (records not available for review). At the time of her most recent genetics evaluation, her height was 5′3.75″ with a BMI of 37.89 kg/m^2^. Medical history was notable for irritable bowel syndrome, ovarian cysts, migraines, joint laxity, fibromyalgia, carpal tunnel syndrome, and plantar fasciitis.

A custom multigene panel including next-generation sequencing with copy number variation analysis of genes *ABCC8*, *APPL1*, *BLK*, *CEL*, *CEP19*, *DYRK1B*, *GATA6*, *GCK*, *GLUD1*, *GNAS*, *HADH*, *HNF1A*, *HNF1B*, *HNF4A*, *INS*, *KCNJ11*, *KLF11*, *KSR2*, *LEP*, *LEPR*, *MC4R*, *NEUROD1*, *NR0B2*, *NTRK2*, *PAX4*, *PCSK1*, *PDX1*, *POMC*, *PPARG*, *RFX6*, *SH2B1*, *SIM1,* and *UCP3* in addition to mitochondrial genome analysis identified a heterozygous variant of uncertain significance in the *GCK* gene (c.679 + 3A > T, splice site). Pathogenic variants in the *GCK* gene are associated with *GCK*-MODY. The patient became pregnant, and her diabetes was well-controlled within guidelines throughout her pregnancy. She is currently participating in the Chicago Monogenic Diabetes Registry.

#### FPLD type 2—*LMNA* (c.717C > A, p.Ser239Arg)

A 29-year-old female presented with body habitus suggestive of FPLD with muscular extremities and increased adipose tissue in her trunk. She had classical phlebomegaly and mons pubis fat hypertrophy characteristic of Dunnigan syndrome. Her mother, who was also seen with her, had a similar phenotype. She noticed more hair growth than her peers during puberty. She had a fatty liver on prior imaging scans, though most recent scans prior to her genetics evaluation were normal with normal LFTs. She had a history of elevated cholesterol and triglycerides. Additionally, she had a history of a high-grade dysplastic adenoma and a juvenile polyp.

A custom multigene panel including next-generation sequencing with copy number variation analysis of genes *AGPAT2*, *AKT2*, *APC*, *AXIN2*, *BANF1*, *BLM*, *BMPR1A*, *BSCL2*, *CAV1*, *CDH1*, *CIDEC*, *EPCAM*, *FBN1*, *GALNT12*, *GREM1*, *KCNJ6*, *LIPE*, *LMNA*, *MFN2*, *MLH1*, *MLH3*, *MSH2*, *MSH3*, *MSH6*, *MUTYH*, *NTHL1*, *PCYT1A*, *PIK3R1*, *PLIN1*, *PMS2*, *POLD1*, *POLE*, *PPARG*, *PSMA3*, *PSMB4*, *PSMB8*, *PSMB9*, *PTEN*, *PTRF*, *RPS20*, *SMAD4*, *SPRTN*, *STK11*, *TBC1D4*, *TP53*, *WRN*, and *ZMPSTE24* identified a heterozygous variant of uncertain significance in the *LMNA* gene (c.717C > A, p.Ser239Arg). Clinical genetic testing through a second laboratory confirmed the presence of the variant, also classified as a variant of uncertain significance. Pathogenic variants in the *LMNA* gene are associated with FPLD type 2. Testing of family members with histories of similar body habitus is being coordinated to contribute evidence towards possible variant reclassification.

#### FPLD type 3—*PPARG* gene (c.610T > C, p.Ser204Pro)

A 40-year-old female presented with prominent muscularity and minimal fat in her extremities in her early 30s following pregnancy. She had 3 episodes of acute pancreatitis over the course of 6 months at age 37 attributed to hypertriglyceridemia. She had been followed for prediabetes beginning at age 25 and had mild acanthosis nigricans. Medical history was otherwise normal for hypertension, ovarian cysts, nonalcoholic fatty liver disease at age 35, irritable bowel syndrome, gastroesophageal reflux disease, gastric ulcers, hiatal hernia, papillary thyroid cancer at age 36, and vestibular migraines.

Genetic testing ordered by an outside provider, which included analysis of genes *ADRA2A, AGPA2, AKT2, BSCL2, CAV1, CAVIN1, CIDEC, FBN1, KCNJ6, LIPE, LMNA, LMNB2, PCYT1A, PIK3R1, PLIN1, POLD1, PPARG, SMB8,* and *ZMPSTE24*, identified a heterozygous variant of uncertain significance in the *PPARG* gene (c.610T > C, p.Ser204Pro). Pathogenic variants in the *PPARG* gene are associated with FPLD type 3. She also participated in a clinical trial for FPLD.

### Cases Without a Diagnosis

In the cohort, 62 patients did not receive a definitive genetic diagnosis for their endocrinologic condition (mean age 41 years, range 18-78; 44 assigned female at birth). Suspected diagnoses evaluated by genetic testing included monogenic obesity (28), lipodystrophy (25), MODY (15), and dyslipidemia (7). Nine underwent whole-exome sequencing as a first-line test, and 3 additional patients underwent whole-exome sequencing as a second-line test following a nondiagnostic multigene panel or chromosomal microarray; the remaining 52 had a nondiagnostic multigene panel, and 1 of these individuals also had a normal chromosomal microarray performed concurrently with the multigene panel. Out of these cases, 15 (24.2%) had at least 1 variant of uncertain significance on genetic testing ordered to assess their endocrinologic condition. Many patients with nondiagnostic results and who had a clinical diagnosis of diabetes were then referred to the ongoing RADIANT study designed to study patients with rare and atypical forms of diabetes [41] or other external endocrinology-specific research programs. Interesting cases sparked multidisciplinary discussion and/or internal research endeavors. Based on availability, some were enrolled in clinical trials of novel treatment relevant to the clinical presentations or challenging clinical circumstances.

## Discussion

This study reports genetics evaluation outcomes and highlights clinical features of patients evaluated in an ADG clinic, part of a multidisciplinary Atypical Diabetes Program collaboration with endocrinologists, aiming to streamline the clinical care for individuals with suspected monogenic conditions predisposing to the development of diabetes. Recognition of patient clinical characteristics that aligned with a potential genetic etiology, informed by endocrinology provider experience, played a key role in initiating genetics referral.

Definitive diagnosis of a monogenic endocrinological condition can inform medical management, which may require multidisciplinary subspecialty care, including and beyond their metabolic concerns. In patients with confirmed genetic lipodystrophy, for example, individuals with pathogenic variants in the *LMNA* gene should engage in close cardiac follow-up given the high reported risk for cardiac disease, especially arrhythmias [12], while those with pathogenic variants in the *MFN2* gene are expected to have neuropathy and should undergo neurologic evaluation [42]. Metreleptin has been approved for the management of FPLD in Europe and Japan, and clinical trials available globally are exploring use of this medication and other analogs [5, 43]. In patients with confirmed MODY, certain subtypes have specific treatment recommendations, such as the use of sulfonylureas, no pharmacologic intervention necessary, or need for multidisciplinary care given multisystem features [13]. Advancing clinical trials eventually leading to Food and Drug Administration-approved treatments only strengthens the need for genetic diagnoses in these patients.

In the current patient cohort, diagnostic genetic testing was highest for individuals with suspected lipodystrophy, with 4 (13.8%) individuals receiving diagnostic results, whereas only 1 individual each received a confirmed genetic diagnosis for monogenic diabetes or monogenic obesity. This is potentially attributed to various factors: overlap in symptom presentation with conditions of multifactorial etiology, referral bias with some endocrinology providers ordering sponsored or research-based genetic testing for most patients to allow more challenging cases access to limited genetics appointment slots, and/or increased access to genetics evaluations given lack of stringent criteria for referral or genetic testing. Despite the relatively low diagnostic yield, the clinical impact of precise treatment for patients should not be overlooked, and even several of those without a genetic diagnosis became eligible for future research involvement. The Atypical Diabetes Program's broad inclusion criteria is a strength in that it allows for genetic exploration of cases that do not meet syndrome-specific clinical guidelines or definitions and therefore may be ineligible for participation through external programs. Continued experience with the Atypical Diabetes Program and its outcomes will inform refinement of its structure and evaluation strategies.

Nearly a quarter (22.0%) of all patients were identified to have at least 1 variant of uncertain significance. This a gene variant for which there is currently insufficient evidence (ie, based on multiple criteria such as presence in individuals with a consistent phenotype vs controls, algorithmic or functional predictions, etc.) to determine whether it has a harmful or neutral effect on the associated protein structure or function. Previous research shows that if reclassified, most variants of uncertain significance are reclassified to benign or likely benign, not associated with disease [44]. These gene variants may warrant future investigation (ie, with functional validation studies) to determine if they can be reclassified; the provision of detailed phenotypes for patients with a variant of uncertain significance through this work may contribute evidence toward future reclassification.

Previous studies have explored the efficacy and strategies of genetic testing for heritable conditions that predispose to diabetes. A multi-institutional systematic review with international collaborators has recommended clinical guidelines for identification of individuals to consider testing for monogenic diabetes and included incorporating a genetic counselor as part of the care team among recommendations [34]. Participants in the current study received both pretest and posttest genetic counseling, which enabled a detailed discussion of the benefits and limitations of genetic testing as well as the nuances of results. The National Society of Genetic Counselors published a practice resource for diabetes mellitus in August 2023 with the goal of increasing genetic counselors’ awareness of the existence of monogenic diabetes and knowledge of implications of a diagnosis, as well as to empower providers with guidance in counseling and identifying genetic testing strategies [30]. One previous study described a proposed model for integrating diabetes genetic diagnosis into routine diabetes care with recruitment through a screening questionnaire, provider identification, or self-referral, the latter being exceptionally efficient in identifying individuals with suspicious features for a monogenic component to their diabetes [35]. Cost-effectiveness analyses have previously shown that genetic testing for monogenic diabetes can save downstream healthcare costs, indicating that coverage by health insurance companies of this testing could both improve timely access to diagnosis and treatment as well as contribute to saving public health dollars [45]. Embedding specific genetics expertise in highly specialized clinics can increase the ease of genetic testing earlier in evaluation and treatment.

Strengths of this study include that it has yielded detailed phenotyping of patients evaluated through the multidiscplinary collaboration between genetics providers and endocrinologists at a large tertiary referral medical center with expertise in lipodystrophy, MODY, and other heritable conditions predisposing to diabetes. As this collaboration hosts both national and international experts in these conditions, the participants in the Atypical Diabetes Program had access to novel clinical trials and studies. Limitations of this work include a relatively small number of patients evaluated to date. Additionally, for some rare endocrinologic disorders, consensus criteria for consideration of genetics evaluation and/or genetic testing do not exist, thus potentially contributing to a lower diagnostic yield. Future studies can build on these findings with larger cohorts of patients and/or with tentative genetics referral and/or testing criteria to clarify yield estimates as well as patient factors that increase the diagnostic yield. Alternative models aside from initial endocrinology evaluation followed by genetics referral and evaluation may also be explored.

The multidisciplinary collaboration between providers and endocrinologists within the Atypical Diabetes Program enabled streamlined clinical care and conclusion of the diagnostic odyssey for patients with a heritable condition predisposing to diabetes. This work details the clinical features that initially prompted suspicion for a genetic etiology, to which endocrinologists may benefit from increased attention in practice; these conditions are highly actionable given that treatment and/or clinical trial eligibility is often informed by genetic status and that a diagnostic test result enables cascade screening for relatives. Early diagnosis can facilitate speedy intervention to reduce endocrine comorbidities and provide appropriate treatment, which ultimately can be life-saving.

## Data Availability

Restrictions apply to the availability of some or all data generated or analyzed during this study to preserve patient confidentiality. The corresponding author will, on request, detail the restrictions and any conditions under which access to some data may be provided.
